# Revisits, Readmission, and Mortality From Emergency Department Admissions for Older Adults With Vague Presentations: Longitudinal Observational Study

**DOI:** 10.2196/55929

**Published:** 2025-02-06

**Authors:** Sebastian Alejandro Alvarez Avendano, Amy Cochran, Valerie Odeh Couvertier, Brian Patterson, Manish Shah, Gabriel Zayas-Caban

**Affiliations:** 1 Department of Industrial and Systems Engineering, College of Engineering, University of Wisconsin-Madison, 3107 Mechanical Engineering Building, 1513 University Avenue, Madison, WI, 53706, United States, 1 (608) 262-2686; 2 Population Health Sciences, School of Medicine and Public Health, University of Wisconsin–Madison, Madison, WI, United States; 3 BerbeeWalsh Department of Emergency Medicine, School of Medicine and Public Health, University of Wisconsin-Madison, Madison, WI, United States

**Keywords:** gerontology, geriatric, older adults, elderly, older people, aging, emergency department, emergency room, ED, disposition decision, disposition, discharge, admission, revisit, readmission, observational study, health, hospital

## Abstract

**Background:**

Older adults (65 years and older) often present to the emergency department (ED) with an unclear need for hospitalization, leading to potentially harmful and costly care. This underscores the importance of measuring the trade-off between admission and discharge for these patients in terms of patient outcomes.

**Objective:**

This study aimed to measure the relationship between disposition decisions and 3-day, 9-day, and 30-day revisits, readmission, and mortality, using causal inference methods that adjust for potential measured and unmeasured confounding.

**Methods:**

A longitudinal observational study (n=3591) was conducted using electronic health records from a large tertiary teaching hospital with an ED between January 1, 2014 and September 27, 2018. The sample consisted of older adult patients with 1 of 6 presentations with significant variability in admission: falls, weakness, syncope, urinary tract infection, pneumonia, and cellulitis. The exposure under consideration was the ED disposition decision (admission to the hospital or discharge). Nine outcome variables were considered: ED revisits, hospital readmission, and mortality within 3, 9, and 30 days of being discharged from either the hospital for admitted patients or the ED for discharged patients.

**Results:**

Admission was estimated to significantly decrease the risk of an ED revisit after discharge (30-day window: −6.4%, 95% CI −7.8 to −5.0), while significantly increasing the risk of hospital readmission (30-day window: 5.8%, 95% CI 5.0 to 6.5) and mortality (30-day window: 1.0%, 95% CI 0.4 to 1.6). Admission was found to be especially adverse for patients with weakness and pneumonia, and relatively less adverse for older adult patients with falls and syncope.

**Conclusions:**

Admission may not be the safe option for older adults with gray area presentations, and while revisits and readmissions are commonly used to evaluate the quality of care in the ED, their divergence suggests that caution should be used when interpreting either in isolation.

## Introduction

Care for acute illnesses has shifted from outpatient offices to emergency departments (EDs), leading to an increase in ED use that has outpaced population growth [1]. EDs diagnose and treat acute illnesses [2]. Therefore, emergency providers diagnose patients, initiate treatment, and predict the disease trajectory to decide whether to admit the patient to an inpatient unit or discharge the patient home [3,4]. Any change to admission decisions can impact outcomes and costs since two-thirds of ED health care costs in the United States come from visits that end in admission [5,6].

Whether to admit or discharge a patient is a weighty decision. This point is best exemplified with older adults (≥65 years of age). Discharging an older adult carries a high risk of adverse health outcomes, especially when compared with younger patients [7-12]. Hence, increasing interest has been placed on identifying patients at risk for adverse outcomes after ED discharge [7,11,13,14] or on developing strategies for following up on discharged patients [15]. Admitting a patient who can be discharged carries its own risks. Older adults are vulnerable to deconditioning and hospital acquired infection, as well as developing delirium and accelerated cognitive decline [16-19]. These issues underscore the importance of measuring the trade-off between admitting and discharging a patient.

Admission decisions are usually based on well-defined clinical factors and practice guidelines, but often, patients fall into a gray area in which the need for hospital admission is unclear based on objective information or even local standards of care. A large group of such gray-area patients is those presenting with syndromic diagnoses such as falls [20] or weakness [21], or patients presenting with more definite diagnoses that are associated with wide variability in admission decision, such as syncope [22], chest pain [5,23,24], urinary tract infection (UTI) [25], pneumonia [26], and cellulitis [27]. Moreover, other factors influence disposition decisions including triage [28], crowding [29], patient home environment [30], diagnostic testing [31], patient ethnicity [32], and hospital capacity [32]. Consequently, admission rates vary widely for these patients between providers and between hospitals, leading to potentially harmful practice variation [33]. Reducing this variation may avoid harmful admissions while safeguarding patient safety, but in order to do so evidence must be used to guide decision-making.

In this study, we focused on older adults in the ED with gray-area diagnoses as follows: diagnoses associated with clinical ambiguity or high rates of potentially preventable hospitalizations and variability in admissions [16,17]. The goal of this work was to identify how the decision to admit drives subsequent revisit, readmission, and mortality among older adult patients with diagnoses that are syndromic (falls and weakness) or lacking a clear standard of practice (syncope, UTI, pneumonia, or cellulitis). This question was difficult to answer since patients with different disposition decisions differ in their clinical severity, complexity, and needs. Without adjusting for these differences, unfair conclusions can be drawn if patients with different disposition decisions are directly compared. We thus used a causal inference methodology for observational data to measure the relationship between disposition decisions and outcomes for these older adult patients [34,35].

## Methods

### Data

ED visits were analyzed using electronic health records (EHRs) from a large Midwestern academic health system. The dataset used was from a common EHR system, and the analyzed population consisted of encounters with a specific large ED between January 1, 2014 and September 27, 2018. ED visits were included for older adult patients (65 years of age or older) with presentations for falls (n=1581), weakness (n=564), syncope (n=468), UTI (n=456), pneumonia (n=299), and cellulitis (n=223). For all 6 presentations, inclusion and exclusion criteria were specified to capture patients who, based on objective criteria present in the EHR occupied a “gray area” with regards to criteria for admission. In general, the strategy was to include patients who met diagnostic criteria for each presentation but did not have further abnormalities indicating a clear indication for admission present within discrete fields of the EHR. General inclusion criteria were an acuity level (emergency severity index; ESI) of 2 or 3, excluding visits assigned the most and least severe ESI levels; and treated and either admitted to the hospital or discharged to their residence, excluding patients who eloped, left against medical advice, or were transferred to another facility. General exclusion criteria were a missing disposition decision, a visit in the last 45 days of the sample period (as these patients had inadequate follow-up for outcomes), indicators of acute coronary syndromes (troponin levels >0.10 ng/mL), severe vital sign abnormalities at any point during the ED visit (systolic blood pressure <80 mmHg, respiration rate >30 BPM, pulse oximetry <88%, or heart rate >120 BPM), or a specific diagnosis that unambiguously required admission (ie, stroke, myocardial infarction, or femur fractures). In addition, patients presenting to the ED with a combination of cellulitis and a higher temperature than 100.3 F were eliminated from the analysis since these encounters had a high admission probability.

### Variables

Seven variables represented baseline characteristics as follows: age, sex, insurance, history of diabetes, history of congestive heart failure, history of hypertension, and Centers for Medicare and Medicaid Services Hierarchical Condition Category (HCC) score [36]. HCC scores predict health care costs and outcomes by assigning risk weights to comorbidities based on ICD codes; higher scores indicate a greater predicted risk and resource use. These baseline characteristics were selected because they are available prior to the ED visit and can capture key clinical variations that could influence a patient’s ED visit and outcomes.

Five variables represented initial observations serving as a proxy for underlying latent health state at the start of the admission process: acuity, temperature, blood pressure, respiratory rate, and heart rate. Acuity was measured using the ESI level, a 5-level triage system where lower ESI levels (eg, 1 or 2) indicate higher acuity, meaning the patient requires more urgent care and significant resources. These proxy variables were included because they are available before the admission process begins and can be considered indirect (or “noisy”) measurements of the patient’s latent health state.

Two variables represented the admission process: treatment time (duration between when a patient is placed in an ED room after triage for treatment and when their admission decision is made) and admission decision. Patients discharged to a Skilled Nursing Facility or Inpatient Rehabilitation Facility were treated as a discharge.

Three primary outcome variables were considered: ED revisits, hospital readmission, and mortality within 30 days of being discharged from either the hospital for admitted patients or the ED for discharged patients. Binary variables for revisits indicated whether an individual returned to the study ED (based on EHR data) within 30 days of discharge. Similarly, binary variables for readmission and mortality indicated whether an individual was readmitted or died within 30 days of discharge, respectively. Alternative windows (3 d and 9 d instead of 30 d) were also considered as secondary outcomes, since a short window is likely to capture only subsequent events related to the original ED visit, whereas a long window is likely to capture all subsequent events related to the original ED visit. To optimize this trade-off, a 9-day window has previously been recommended [37].

Missing continuous and categorical variables were imputed with the median and most frequent category, respectively. Continuous variables were standardized and these transformed variables were then used for estimation.

### Analysis

Admission decisions were evaluated using 3 types of analyses: unadjusted, adjusted, and subgroup. Each type of analysis was repeated for each of the 3 primary outcomes, and for the overall sample and each presentation group. Unadjusted analyses involved estimating the observed (ie, unadjusted) difference in risk of an outcome between admitted patients and discharged patients. Wald 95% CIs were recovered.

Adjusted analyses, constituting our primary analyses, involved estimating these same risk differences (RDs) but adjusted for the unmeasured or latent health state in addition to measured variables (ie, baseline characteristics and proxy variables). These estimates were recovered by implementing a latent-variable approach for evaluating admission decisions (see Cochran et al [34] for details and applied in Alvarez Avendaño et al [35] to chest pain patients). Briefly, this approach involves modeling both the measured variables and a latent “health state” variable, and then fitting this model to data using expectation maximization, from which an estimate of the average treatment effect (ie, average difference in potential outcomes were we to admit vs discharge a patient) and Wald CIs can be recovered. The latent health state is included to account for potential unmeasured confounding between the admission decision and each outcome. More specifically, it accounts for confounding by indication, whereby admission decisions are based on factors not captured in the data. The inclusion of proxy variables, such as acuity and vitals, is crucial as they provide indirect measures of the latent health state, thereby strengthening the model’s ability to mitigate confounding by indication.

The model comprised several regression components: logistic regression to model the latent health state as a function of baseline characteristics; logistic and linear regression to model the proxy variables (eg, acuity and vitals) as a function of latent health and baseline factors; threshold regression to model the admission process (decision and timing) as a function of latent health, baseline characteristics, and proxy variables; and linear regression to model each outcome based on latent health. Once model parameters are estimated, we calculate average treatment effects by comparing outcomes for each visit under hypothetical admission and discharge scenarios while holding all other variables at their original value. We then calculate the difference between these hypothetical outcomes and average this difference across all visits to obtain the overall treatment effect. For subgroup analyses, we recover subgroup-specific average treatment effects by averaging these hypothetical outcomes across only those visits with certain baseline characteristics (eg, female patients).

To check the sensitivity of our conclusions to various factors, several additional analyses were conducted. First, we checked sensitivity to 2 key assumptions for the latent-variable approach, which is that potential outcomes are independent of admission decisions with similar latent health needs and baseline characteristics and that events related to different ED visits are independent. Second, we analyzed the secondary outcomes, which use alternative time windows (3 days and 9 days instead of 30 days). Third, we estimated the same RDs as the main analyses, but only adjusted for the measured variables as opposed to both the measured variables and the latent health state. These estimates were recovered using the causal inference methods known as inverse probability weighting (IPW) and g-estimation.

Due to space considerations, sensitivity analyses are detailed in Multimedia Appendix 1. Importantly, estimates from checking violations to our 2 key assumptions were generally consistent in terms of direction and magnitude with the adjusted estimates reported in the main text, and estimates from IPW and g-estimation were similarly consistent with unadjusted estimates, with a few exceptions detailed in Multimedia Appendix 1. In addition, cellulitis had a small sample size (n=223) relative to other diagnostic groups, leading to imprecise estimates. Therefore, results for cellulitis are presented in Multimedia Appendix 1. Finally, subgroup analyses for individual diagnoses and technical details of all our adjusted analyses can also be found in Multimedia Appendix 1.

Significance was considered at an α level of 0.05. Hypothesis tests were 2-tailed Wald tests. Multiple comparisons were not adjusted for, and as such, nominal CIs and *P* values are reported.

#### Ethical Considerations

This study was reviewed by the UW Minimal Risk Research Institutional Review Board (ID 2024-0106-CP001) and was deemed to meet the federal criteria for exemption.

## Results

### Baseline Characteristics

Sample characteristics are summarized in Table 1. Patients were predominantly female (2102/3591, 58.5%) with Medicare insurance (1460/1589, 91.9%) and had an average age of 79 years. Patients were diagnosed with falls (1581/3591, 44%), weakness (564/3591, 16%), syncope (468/3591, 13%), UTI (456/3591, 13%), pneumonia (299/3591, 8%), and cellulitis (223/3591, 6%).

**Table 1.** Descriptive statistics of sample by complaint.

	All(n=3591)	Falls(n=1581)	Weakness(n=564)	Syncope(n=468)	UTI^a^(n=456)	Pneumonia(n=299)	Cellulitis(n=223)
Age (years), mean (SD)	79.23 (8.93)	80.56 (8.94)	78.66 (8.4)	76.8 (8.73)	79.22 (8.91)	78.9 (9.27)	76.758 (8.36)
Comorbidity (HCC^b^), mean (SD)	1.65 (1.41)	1.49 (1.22)	1.82 (1.46)	1.32 (1.36)	1.879 (1.6)	2.107 (1.57)	1.965 (1.72)
Heart rate (BPM), mean (SD)	78.07 (14.66)	76.41 (13.54)	78.39 (15.06)	73.11 (13.52)	80.79 (14.82)	87.32 (15.74)	81.41 (14.59)
Temperature (°F), mean (SD)	97.61 (1.4)	97.44 (1.69)	97.56 (0.85)	97.34 (0.72)	97.88 (1.18)	98.55 (1.56)	97.73 (0.8)
Blood pressure (mmHg), mean (SD)	74.2 (13.7)	76.23 (13.92)	74.36 (12.9)	71.91 (12.19)	73.49 (14.72)	69.47 (13.57)	71.99 (12.41)
Respiration rate (BPM), mean (SD)	18.07 (3.18)	17.82 (2.92)	17.86 (3.23)	17.72 (3.57)	18.28 (3.07)	19.8 (3.72)	18.41 (2.58)
Treatment time (hours), mean (SD)	1.82 (0.12)	0.18 (0.1)	0.2 (0.12)	0.22 (0.2)	0.16 (0.07)	0.13 (0.06)	0.15 (0.08)
Female, n (%)	2102 (58.5)	994 (62.9)	299 (53)	255 (54.5)	290 (63.6)	149 (49.8)	115 (51.6)
Insurance, n (%)							
Medicaid/Badger Care	15 (0.4)	3 (0.2)	3 (0.5)	5 (1.1)	2 (0.4)	1 (0.3)	1 (0.4)
Medicare	3278 (91.9)	1460 (92.9)	526 (93.6)	415 (90.8)	409 (90.1)	273 (91.3)	195 (87.4)
Commercial/Worker’s Compensation	271 (7.6)	109 (6.9)	33 (5.9)	35 (7.7)	42 (9.3)	25 (8.4)	27 (12.1)
Self-pay	3 (0.1)	0 (0)	0 (0)	2 (0.4)	1 (0.2)	0 (0)	0 (0)
Diabetes, n (%)	744 (20.7)	293 (18.5)	138 (24.5)	81 (17.3)	99 (21.7)	72 (24.1)	61 (27.4)
Congestive Heart failure, n (%)	413 (11.5)	173 (10.9)	62 (11)	33 (7.1)	56 (12.3)	53 (17.1)	36 (16.1)
Hypertension, n (%)	2081 (58.0)	923 (58.4)	350 (62.1)	244 (52.1)	255 (55.9)	170 (56.9)	139 (62.3)
Acuity (=2), n (%)	993 (27.7)	425 (26.9)	139 (24.6)	195 (41.7)	103 (22.6)	106 (35.5)	25 (11.2)
Admitted, n (%)	1401 (39.0)	333 (21.1)	252 (44.7)	179 (38.2)	240 (52.6)	255 (85.3)	142 (63.7)
30-day revisits, n (%)	644 (17.9)	293 (18.5)	108 (19.1)	53 (11.3)	105 (23)	40 (13.4)	45 (20.2)
30-day readmission, n (%)	207 (5.8)	50 (3.2)	36 (6.4)	23 (4.9)	43 (9.4)	33 (11)	22 (9.9)
30-day mortality, n (%)	127 (3.5)	50 (3.2)	30 (5.3)	6 (1.3)	11 (2.4)	27 (9)	3 (1.3)

a UTI: urinary tract infection.

b HCC: hierarchical condition category.

### Revisits

Table 2 summarizes the unadjusted RD between admission and discharge for 30-day revisits across all presentations and for individual presentations. Across all presentations, admission carried a significantly lower unadjusted risk than a discharge of 30-day revisits (RD=−5.2%, 95% CI −6.3 to −4.1). Individual presentations yielded unadjusted estimates that generally agreed with that of the entire sample, with 1 exception. For syncope patients, admission carried a greater unadjusted risk than a discharge of 30-day revisits (RD=2.5%, 95% CI 0.0 to 5.0).

**Table 2.** Unadjusted and adjusted estimates (95% CI) of risk differences (RD) for 30-day revisits, comparing admission to discharge (reference). Adjusted estimates account for latent health state and measured variables.

	Unadjusted RD (95% CI)	Adjusted RD (95% CI)
All	−5.2 (−6.3 to −4.1)	−6.4 (−7.8 to −5.1)
Falls	−4.5 (−6.5 to −2.5)	−33.2 (−35.5 to −30.8)
Weakness	−8.8 (−11.6 to −6.0)	4.4 (−2.8 to 11.6)
Syncope	2.5 (0 to 5)	−38.6 (−43.2 to −34.1)
UTI^a^	−10.8 (−14.1 to −7.5)	−56.7 (−61.3 to −52.2)
Pneumonia	−3.0 (−7.6 to 1.7)	23.4 (18.7 to 28.1)

a UTI: urinary tract infection.

Table 2 also summarizes the estimated RDs, adjusted for latent health state and measured patient variables, between admission and discharge for 30-day revisits. Across all presentations, admission carried a significantly lower adjusted risk than a discharge of 30-day revisits (RD=−6.4%, 95% CI −7.8 to −5.0). Individual presentations yielded adjusted estimates that generally agreed with that of the entire sample, with the following exceptions. For patients with weakness, admission carried a numerically greater adjusted risk than a discharge of 30-day revisits (RD=4.4%, 95% CI −2.8 to 11.6). For patients with pneumonia, admission carried a significantly greater adjusted risk than a discharge of 30-day revisits (RD=23.4%, 95% CI 18.7 to 28.1).

### Readmission

Table 3 summarizes the unadjusted RD between admission and discharge for 30-day readmissions across all diagnoses and for individual diagnoses. Across all diagnoses, admission carried a significantly greater unadjusted risk than a discharge of 30-day readmission (RD =14.8%, 95% CI 14.1 to 15.4). Individual diagnoses yielded unadjusted estimates that agreed in terms of direction, significance, and magnitude (within 3%) with that of the entire sample.

**Table 3.** Unadjusted and adjusted estimates (95% CI) of risk differences (RDs), in percentage points, for 30-day readmissions, comparing admission to discharge (reference). Adjusted estimates account for latent health state and measured variables.

	Unadjusted RD (95% CI)	Adjusted RD (95% CI)
All	14.8 (14.1 to 15.4)	5.8 (5.0 to 6.5)
Falls	15.0 (14.1 to 15.9)	3.2 (2.3 to 4.0)
Weakness	14.3 (12.6 to 16.0)	61.6 (57.7 to 65.5)
Syncope	12.8 (11.1 to 14.6)	4.9 (3.0 to 6.9)
UTI^a^	17.9 (15.6 to 20.2)	9.4 (6.8 to 12.1)
Pneumonia	12.9 (8.7 to 17.2)	25.8 (20.9 to 30.6)

a UTI: urinary tract infection.

Table 3 also summarizes estimated RDs, adjusted for latent health state and measured patient variables, between admission and discharge for 30-day readmissions. Across all diagnoses, admission carried a significantly greater adjusted risk than a discharge of 30-day readmission (RD=5.8%, 95% CI 5.0 to 6.5). Individual diagnoses yielded adjusted estimates in the same direction as that of the entire sample, but the magnitude was notably larger in a few cases. For patients with weakness, admission carried a greater adjusted risk than a discharge 30-day readmission (RD=61.6%, 95% CI 57.7 to 65.5). For patients with pneumonia, admission carried a significantly greater adjusted risk than a discharge of 30-day readmission (RD=25.8%, 95% CI [20.9, 30.6]).

### Mortality

Table 4 summarizes the unadjusted RD between admission and discharge for 30-day mortality across all diagnoses and for individual diagnoses. Across all diagnoses, admission carried a significantly greater unadjusted risk than a discharge of 30-day mortality (RD=3.8%, 95% CI 3.3 to 4.3). Individual diagnoses yielded unadjusted estimates that agreed in terms of direction, significance, and magnitude (within 3%) with those of the entire sample.

**Table 4.** Unadjusted and adjusted estimates (95% CI) of risk differences (RDs), in percentage points, for 30-day mortality, comparing admission to discharge (reference). Adjusted estimates account for latent health state and measured patient variables.

	Unadjusted RD (95% CI)	Adjusted RD (95% CI)
All	3.8 (3.3 to 4.3)	1.0 (0.4 to 1.6)
Falls	2.8 (1.9 to 3.7)	−0.9 (−1.8 to 0.0)
Weakness	6.9 (5.3 to 8.5)	58.1 (54.1 to 62.0)
Syncope	2.4 (1.6 to 3.3)	−21.9 (−53.6 to 9.7)
UTI^a^	1.9 (0.7 to 3.1)	1.1 (−0.3 to 2.5)
Pneumonia	5.3 (1.4 to 9.2)	7.7 (4.4 to 11.0)

a UTI: urinary tract infection.

Table 4 also summarizes estimated RDs, adjusted for latent health state and measured patient variables, between admission and discharge for 30-day mortality. Across all diagnoses, admission carried a significantly greater adjusted risk than a discharge of 30-day mortality (RD=1.0%, 95% CI 0.4 to 1.6). Individual diagnoses yielded adjusted estimates differing from that of the entire sample in several meaningful ways.

Reporting from high to low, admission for patients with weakness carried a significantly greater adjusted risk than a discharge of 30-day mortality (RD=58.1%, 95% CI 54.1 to 62.0). For patients with pneumonia, admission also carried a significantly adjusted risk than a discharge of 30-day mortality (RD=7.7%, 95% CI 4.4 to 11.0). For patients with UTI, admission carried a numerically greater adjusted risk than a discharge of 30-day mortality (RD=1.1%, 95% CI −0.3 to 2.5). For patients with syncope, admission carried a numerically lower adjusted risk of 30-day mortality (RD=−21.9%, 95% CI −53.6 to 9.7). For patients with falls, admission carried a lower adjusted risk than a discharge of 30-day mortality (RD=−0.9%, 95% CI −1.8 to 0.0).

### Subgroup Analyses

Table 5 summarizes subgroup-specific estimates of RDs, adjusted for latent health state and measured patient variables, between admission and discharge for 30-day outcomes. All subgroup estimates had the same direction as that of the entire sample: negative for 30-day revisits and positive for 30-day readmission and mortality. For all 30-day outcomes, 5 subgroups had numerically greater adjusted estimates than that of the entire sample. These subgroups (in decreasing order of prominence) were individuals with congenital heart disease, individuals with diabetes, individuals with HCC scores greater than the mean score of 1.65, individuals greater than the mean age of 79.2 years, and individuals with hypertension. Of note, all these subgroups tend to have worse baseline health than their counterpart. The subgroup with the numerically lowest adjusted estimate for all 30-day outcomes was individuals with insurance other than Medicare.

**Table 5.** Subgroup-specific adjusted estimates (95% CI) of risk differences, in percentage points, for 30-day outcomes, comparing admission to discharge (reference). Adjusted estimates account for latent health state and measured patient variables.

Variable and subgroup		Revisit	Readmission	Mortality
Age (years)				
65‐79.2		−7.2 (−8.7 to −5.6)	5.4 (4.7 to 6.2)	0.9 (0.2 to 1.5)
>79.2		−5.6 (−7.0 to −4.1)	6.1 (5.3 to 7.0)	1.2 (0.6 to 1.9)
Sex				
Male		−6.5 (−8.0 to −4.9)	5.8 (5.0 to 6.6)	1.0 (0.4 to 1.7)
Female		−6.4 (−7.9 to −5.0)	5.8 (5.0 to 6.6)	1.0 (0.4 to 1.7)
Insurance				
Medicare		−6.2 (−7.6 to −4.9)	5.8 (5.1 to 6.6)	1.1 (0.5 to 1.7)
Other		−8.5 (−10.8 to −6.3)	4.9 (3.9 to 5.9)	0.6 (−0.2 to 1.3)
Diabetes				
No		−6.8 (−8.2 to −5.4)	5.6 (4.9 to 6.4)	0.9 (0.3 to 1.6)
Yes		−5.0 (−6.7 to −3.2)	6.4 (5.4 to 7.4)	1.4 (0.7 to 2.1)
Congenital heart failure				
No		−7.0 (−8.4 to −5.6)	5.5 (4.8 to 6.2)	0.9 (0.3 to 1.5)
Yes		−1.8 (−3.9 to 0.3)	7.8 (6.6 to 9.0)	2.1 (1.3 to 2.9)
Hypertension				
No		−6.9 (−8.4 to −5.4)	5.6 (4.8 to 6.3)	0.9 (0.3 to 1.5)
Yes		−6.1 (−7.5 to −4.6)	5.9 (5.1 to 6.7)	1.1 (0.5 to 1.7)
Hierarchical condition category				
≤1.65		−7.4 (−8.8 to −6.0)	5.3 (4.6 to 6.1)	0.8 (0.2 to 1.4)
>1.65		−4.7 (−6.2 to −3.2)	6.5 (5.6 to 7.4)	1.4 (0.8 to 2.1)

## Discussion

### Principal Findings

Our goal was to investigate the impact of admitting older adult patients with 6 common but variably managed ED presentations: falls, weakness, syncope, UTI, pneumonia, and cellulitis. In particular, these conditions show considerable variability in admission practices, and understanding their specific outcomes can help clinicians better determine when admission is beneficial or potentially harmful. Using causal inference methods, we analyzed EHR data to compare outcomes such as ED revisits, hospital readmissions, and mortality, measuring these outcomes at 3, 9, and 30 days after discharge. By focusing on this “gray area” where no standard practice exists, we wanted to provide insights that support more consistent and evidence-based admission decisions for these presentations.

Our primary finding is that admission was associated with a significant decrease in the risk of an ED revisit, but with a significant increase in the risk of hospital readmission and mortality. The association with an increased readmission risk was consistent for all individual diagnoses, for all time frames, and for all estimation approaches (unadjusted, IPW, g-estimation, and latent variable as the primary analysis). The associations with a decreased revisit risk and with an increased mortality risk were generally consistent for individual diagnoses, all time frames, and all approaches, with a few exceptions discussed below. These results would suggest that while admission may help prevent immediate returns to the ED, it could lead to worse long-term outcomes for certain patients. This balance of risks highlights the importance of carefully weighing the short-term benefits of admission against the potential for readmission and mortality, especially in older adults with these conditions.

In our primary analysis, admission was associated with an increased revisit risk for patients with weakness and pneumonia, along with a notably higher readmission and mortality risk for these conditions compared with others. This suggests that patients with weakness or pneumonia may be especially vulnerable to poor outcomes from admission. In contrast, admission was associated with a numerically lower 30-day mortality risk for patients with falls or syncope, indicating that these individuals may benefit more from being admitted. These findings can help guide clinicians in prioritizing which patients may require closer monitoring, alternative care options, or potential admission.

Our subgroup analysis identified patient factors that may influence outcomes from hospital admission. Overall, admission appears less favorable for older individuals (>79 years) and those with comorbidities (ie, congenital heart disease, diabetes, high HCC scores, or hypertension). Important factors for admission, however, may vary by specific presentation, as shown in our subgroup analysis in Multimedia Appendix 1. Despite these differences, we found consistent trends across subgroups, suggesting that standardized admission recommendations could potentially be based on the type of presentation alone, rather than on patient-specific factors like age, sex, insurance, or comorbidity.

For comparison, another study examined the impact of admitting older adult patients presenting to the ED with chest pain, another presentation with variable admission practices, and found that admission had a lower readmission risk but an increased mortality risk [35]. This study similarly estimated an increased mortality risk, yet it also found an increase in readmission risk for almost all presentations. The association of admission with both increased and decreased risks for revisits, readmission, and mortality, underscores why standardized care pathways are lacking for these presentations [21]. While our results suggest that, in general, discharge may be associated with lower readmission and mortality risks, discharging an older adult can still carry significant risks. These findings support the need for safe alternatives to hospitalization, such as outpatient care coordination or at-home ambulatory providers [38]. Such alternatives have been shown to effectively reduce hospital readmission rates on older adult patients discharged from the ED [39,40].

### Limitations

A primary limitation of this study is the potential for unmeasured confounding, especially confounding by indication, where treatment decisions are influenced by clinical indicators not fully captured in the EHR. This limitation is significant because it directly impacts our ability to interpret the findings causally. Although we used multiple methods, including a latent variable approach to account for unobserved health status [34], no method can fully guarantee that all confounding is addressed. Consequently, our findings remain subject to potential bias if residual confounding exists. This is particularly relevant for interpreting the effect of admission on outcomes, as patients who are “sicker” based on unmeasured factors are more likely to be admitted, and these same factors could drive poor outcomes. While the latent variable approach yielded estimates generally more favorable to admission—suggesting some adjustment for underlying severity—and results were consistent across different time frames and presentations, caution is warranted in interpreting these effects as causal. This limitation highlights the need for further research to strengthen the causal inference of admission effects on outcomes.

In addition, this was a retrospective, observational study conducted using EHR data from an academic ED in the US Midwest, thereby limiting the generalizability of our findings. Moreover, the resulting patient population for specific presentations after applying our filters was relatively small, leading to nonsignificant estimates. Replicating this analysis in different EDs with larger samples is needed.

### Conclusions

This study is one of the few to quantify the risks and benefits associated with admission decisions for ED presentations associated with unclear need for hospitalization. Existing studies have mainly focused on identifying factors that influence hospital admission of noncritically ill patients, often through physician surveys [41] or identifying risk patterns [33,42,43]. This study is intended to quantify the trade-offs of admission at a population level rather than directly influencing bedside decisions. Our findings indicate that admission is not always the “safe” choice, underscoring the need for further research to establish specific criteria that can better guide admission decisions for older adults in the ED.

## Supplementary material

10.2196/55929 Multimedia Appendix 1 Accompanying appendix reporting (1) IPW and g-estimation results, (2) description of the latent variable approach, (3) 3-day outcomes results, (4) sensitivity analyses, (5) subgroup analyses for individual diagnoses, (6) analysis for cellulitis diagnosis, and (7) analysis for syndromic and no clear standard practice diagnoses.

## Abbreviations

ED: emergency department
EHR: electronic health record
ESI: emergency severity index
HCC: hierarchical condition category
IPW: inverse probability weighting
RD: risk difference
UTI: urinary tract infection
