# Supplementary material for: Revisits, Readmission, and Mortality From Emergency Department Admissions for Older Adults With Vague Presentations: Longitudinal Observational Study
Source: JMIR Aging. 2025 Feb 6;8:e55929. doi: 10.2196/55929 (PMC11825896; doi:10.2196/55929)
Supplement: Multimedia Appendix 1 [file aging-v8-e55929-s001.docx]

**Supplementary material**

**Contents**

[Inverse probability weighting and g-estimation 2](#_Toc181561520)

[*Description of methods* 2](#_Toc181561521)

[*Results* 2](#_Toc181561522)

[*Limitations* 5](#_Toc181561523)

[Latent-variable approach 6](#_Toc181561524)

[3-day and 9-day outcomes 8](#_Toc181561525)

[Sensitivity analyses 10](#_Toc181561526)

[*Violations in conditional exchangeability* 10](#_Toc181561527)

[*Per-patient analysis* 11](#_Toc181561528)

[Subgroup analyses 15](#_Toc181561529)

[Cellulitis 18](#_Toc181561530)

[Syndromic and no clear standard practice diagnoses 21](#_Toc181561531)

[References 22](#_Toc181561532)

# **Inverse probability weighting and g-estimation**

## ***Description of methods***

In addition to the adjusted and unadjusted estimates presented in the main text, we estimated risk difference using two other causal inference methods. The first method was inverse probability weighting (IPW). This method involves performing logistic regression to predict admission decisions from baseline characteristics, acuity, and vitals. The logistic regression model is then used to estimate each visit’s probability of receiving the realized admission decision conditional on baseline characteristics, acuity, and treatment time. A weight is then recovered for each visit by taking the reciprocal of this estimated probability. With these estimates, an estimate for the risk of admission is then estimated by taking the weighted average of outcomes among admitted patients, and similarly, an estimate for the risk of discharge is then estimated by taking the weighted average of outcomes among discharged visits. CIs are obtained using the method described on pages 922–923 in Wooldridge.^1^

The second method is g-estimation. This method involves building a logistic regression model to predict admission decision from baseline characteristics, proxy variables, and a variable Y−cA for a given constant c where Y is the outcome of interest and A is the realized admission decision. The average treatment effect is given by the value of c for which the estimated coefﬁcient for Y−cA in regression is zero. CIs are given as the range of c for which the estimated coefﬁcient for Y−cA is not signiﬁcantly different from zero, as determined by a Wald hypothesis test.

## ***Results***

Estimates from IPW and g-estimation are reported in Table S1. Unadjusted estimates are also provided for comparison; recall that unadjusted estimates are presented in the main text. Estimates from IPW and g-estimation agreed with unadjusted estimates in terms of direction and magnitude (within 3%), with the only exceptions being estimates for pneumonia patients. For these patients, g-estimation generally provided estimates that were more favorable to admission decisions compared to unadjusted estimates; the former provided estimated risk differences that were roughly 4% lower than the corresponding unadjusted estimate. For these patients, IPW also provided estimates that differed by more than 3% than the unadjusted estimates: 2.1% estimated risk difference for 30-day revisits compared to an -3.0% unadjusted estimate; -4.7% estimated risk difference for 9-day mortality compared to an 1.3% unadjusted estimate; and -1.1% estimated risk difference for 30-day mortality compared to an 5.3% unadjusted estimate. These discrepancies might be a result of a small sample size for pneumonia patients, compared to falls, weakness, syncope, and UTI patients.

**Table S1.** Estimates (95% CI) of adjusted risk differences from IPW and g-estimation.

| **Diagnosis** | **Outcome** | **Unadjusted** | **IPW** | **g-estimation** |
| --- | --- | --- | --- | --- |
| **All** | **3-day Revisit** | -4.7 (-5.3, -4.1) | -4.7 (-5.0, -4.4) | -5.0 (-6.5, -3.6) |
|  | **9-day Revisit** | -5.2 (-6.0, -4.4) | -5.3 (-6.1, -4.5) | -6.0 (-8.1, -4.0) |
|  | **30-day Revisit** | -5.2 (-6.3, -4.1) | -5.4 (-6.8, -3.9) | -6.4 (-9.1, -3.7) |
|  | **3-day Readmission** | 1.6 (1.3, 1.8) | 1.6 (1.5, 1.7) | 1.6 (1.0, 2.1) |
|  | **9-day Readmission** | 6.1 (5.7, 6.6) | 6.3 (5.7, 6.8) | 5.9 (4.8, 7.0) |
|  | **30-day Readmission** | 14.8 (14.1, 15.4) | 14.9 (13.8, 15.9) | 14.4 (12.8, 16.0) |
|  | **3-day Mortality** | 1.8 (1.6, 2.1) | 1.6 (1.5, 1.7) | 1.7 (1.1, 2.3) |
|  | **9-day Mortality** | 2.7 (2.4, 3.1) | 2.2 (2.0, 2.3) | 2.3 (1.4, 3.2) |
|  | **30-day Mortality** | 3.8 (3.3, 4.3) | 2.8 (2.5, 3.2) | 2.9 (1.6, 4.3) |
| **Falls** | **3-day Revisit** | -4.6 (-5.7, -3.6) | -3.7 (-4.4, -2.9) | -4.9 (-7.5, -2.2) |
|  | **9-day Revisit** | -5.5 (-7.0, -4.0) | -5.4 (-6.5, -4.3) | -6.1 (-9.8, -2.5) |
|  | **30-day Revisit** | -4.5 (-6.5, -2.5) | -5.3 (-7.8, -2.8) | -6.0 (-10.9, -1.1) |
|  | **3-day Readmission** | 0.9 (0.7, 1.1) | 1.9 (1.2, 2.7) | 1.1 (0.1, 1.3) |
|  | **9-day Readmission** | 4.8 (4.3, 5.3) | 5.0 (4.1, 6.0) | 4.8 (3.4, 6.3) |
|  | **30-day Readmission** | 15.0 (14.1, 15.9) | 14.4 (12.3, 16.6) | 14.7 (12.5, 16.9) |
|  | **3-day Mortality** | 1.8 (1.5, 2.1) | 1.4 (1.2, 1.6) | 1.7 (0.7, 2.6) |
|  | **9-day Mortality** | 2.4 (1.8, 2.9) | 2.0 (1.5, 2.4) | 2.2 (0.7, 4.3) |
|  | **30-day Mortality** | 2.8 (1.9, 3.7) | 1.7 (0.9, 2.5) | 1.9 (-0.5, 4.3) |
| **Weakness** | **3-day Revisit** | -5.4 (-6.9, -3.9) | -5.6 (-6.6, -4.6) | -5.9 (-6.0, -2.0) |
|  | **9-day Revisit** | -6.3 (-8.5, -4.1) | -7.6 (-10.0, -5.2) | -8.4 (-8.5, -2.9) |
|  | **30-day Revisit** | -8.8 (-11.6, -6.0) | -10.5 (-14.0, -6.9) | -11.5 (-11.7, -4.7) |
|  | **3-day Readmission** | 2.0 (1.3, 2.6) | 1.9 (1.5, 2.4) | 2.1 (2.0, 4.3) |
|  | **9-day Readmission** | 7.1 (5.9, 8.4) | 7.1 (5.8, 8.4) | 7.0 (7.0, 10.2) |
|  | **30-day Readmission** | 14.3 (12.6, 16.0) | 13.6 (11.4, 15.8) | 13.4 (13.3, 17.5) |
|  | **3-day Mortality** | 3.2 (2.3, 4.0) | 3.0 (2.4, 3.5) | 2.9 (-0.0, 5.0) |
|  | **9-day Mortality** | 4.9 (3.7, 6.1) | 4.3 (3.5, 5.1) | 4.4 (4.3, 7.3) |
|  | **30-day Mortality** | 6.9 (5.3, 8.5) | 5.8 (4.5, 7.0) | 5.7 (5.6, 9.6) |

***Table Continued.***

| **Diagnosis** | **Outcome** | **Unadjusted** | **IPW** | **G-estimation** |
| --- | --- | --- | --- | --- |
| **Syncope** | **3-day Revisit** | -1.1 (-2.3, 0.1) | -1.0 (-1.5, -0.5) | -0.8 (-100.0, 3.9) |
|  | **9-day Revisit** | -2.9 (-4.6, -1.1) | -3.6 (-4.5, -2.7) | -3.6 (-3.8, 0.9) |
|  | **30-day Revisit** | 2.5 (-0.0, 5.0) | 0.1 (-2.2, 2.3) | 1.0 (0.8, 5.3) |
|  | **3-day Readmission** | 1.7 (1.0, 2.3) | 1.4 (1.0, 1.8) | 1.4 (-0.0, 3.5) |
|  | **9-day Readmission** | 3.4 (2.5, 4.2) | 2.6 (2.0, 3.2) | 2.9 (-0.0, 5.5) |
|  | **30-day Readmission** | 12.8 (11.1, 14.6) | 10.2 (8.4, 12.1) | 11.3 (-0.0, 15.7) |
|  | **3-day Mortality** | 0.0 (0.0, 0.0) | 0.0 (0.0, 0.0) | 0.0 (0.0, 0.0) |
|  | **9-day Mortality** | 1.1 (0.6, 1.6) | 0.8 (0.6, 1.1) | 1.0 (-0.0, 2.3) |
|  | **30-day Mortality** | 2.4 (1.6, 3.3) | 1.6 (0.9, 2.2) | 1.8 (1.7, 4.5) |
| **UTI** | **3-day Revisit** | -9.5 (-11.4, -7.5) | -9.7 (-11.5, -7.9) | -9.8 (-9.9, -4.7) |
|  | **9-day Revisit** | -9.8 (-12.5, -7.1) | -10.6 (-14.1, -7.1) | -9.3 (-9.5, -2.4) |
|  | **30-day Revisit** | -10.8 (-14.1, -7.5) | -10.4 (-15.7, -5.1) | -9.4 (-9.7, -1.1) |
|  | **3-day Readmission** | 2.1 (1.3, 2.9) | 2.2 (1.8, 2.7) | 2.4 (-0.0, 5.5) |
|  | **9-day Readmission** | 9.2 (7.5, 10.8) | 9.2 (7.8, 10.6) | 9.8 (9.7, 14.0) |
|  | **30-day Readmission** | 17.9 (15.6, 20.2) | 19.1 (16.3, 21.9) | 20.1 (20.0, 25.6) |
|  | **3-day Mortality** | 1.3 (0.6, 1.9) | 0.8 (0.7, 1.0) | 0.6 (-0.0, 2.3) |
|  | **9-day Mortality** | 1.7 (0.9, 2.4) | 1.1 (0.9, 1.2) | 0.8 (-0.0, 2.5) |
|  | **30-day Mortality** | 1.9 (0.7, 3.1) | 1.0 (0.4, 1.6) | 0.7 (0.6, 3.7) |
| **Pneumonia** | **3-day Revisit** | -7.1 (-9.5, -4.8) | -5.1 (-10.5, 0.3) | -8.1 (-8.3, -2.1) |
|  | **9-day Revisit** | -2.8 (-6.2, 0.6) | -1.3 (-6.7, 4.1) | -6.6 (-6.9, 0.5) |
|  | **30-day Revisit** | -3.0 (-7.6, 1.7) | 2.1 (-3.4, 7.6) | -7.2 (-7.5, 4.4) |
|  | **3-day Readmission** | 2.0 (0.2, 3.7) | 1.7 (1.5, 1.9) | 0.6 (-0.0, 3.8) |
|  | **9-day Readmission** | 6.3 (3.2, 9.3) | 5.6 (4.9, 6.2) | 2.0 (1.9, 7.3) |
|  | **30-day Readmission** | 12.9 (8.7, 17.2) | 12.1 (10.6, 13.6) | 8.3 (8.0, 17.2) |
|  | **3-day Mortality** | 0.9 (-1.5, 3.2) | 2.6 (2.1, 3.1) | 2.1 (1.9, 8.4) |
|  | **9-day Mortality** | 1.3 (-1.8, 4.5) | -4.7 (-15.6, 6.2) | -0.2 (-0.4, 7.0) |
|  | **30-day Mortality** | 5.3 (1.4, 9.2) | -1.1 (-12.0, 9.8) | 2.3 (2.0, 11.0) |

## ***Limitations***

For g-estimation and inverse probability weighting to return unbiased estimates of the average causal effect of admission versus discharge, baseline characteristics and proxy variables must comprise all variables that can confound the relationship between the admission decision and the outcome. Other unmeasured factors are suspected which are used by an ED provider to determine their admission decision and which can influence outcomes. This issue is known as confounding by indication. If such an issue is present, as suspected, then g-estimation and IPW will return biased estimates of average causal effects. Therefore, a latent-variable approach, which can adjust for a latent confounder, is also performed and is the primary analysis reported in the main text. This approach is detailed next.

# **Latent-variable approach**


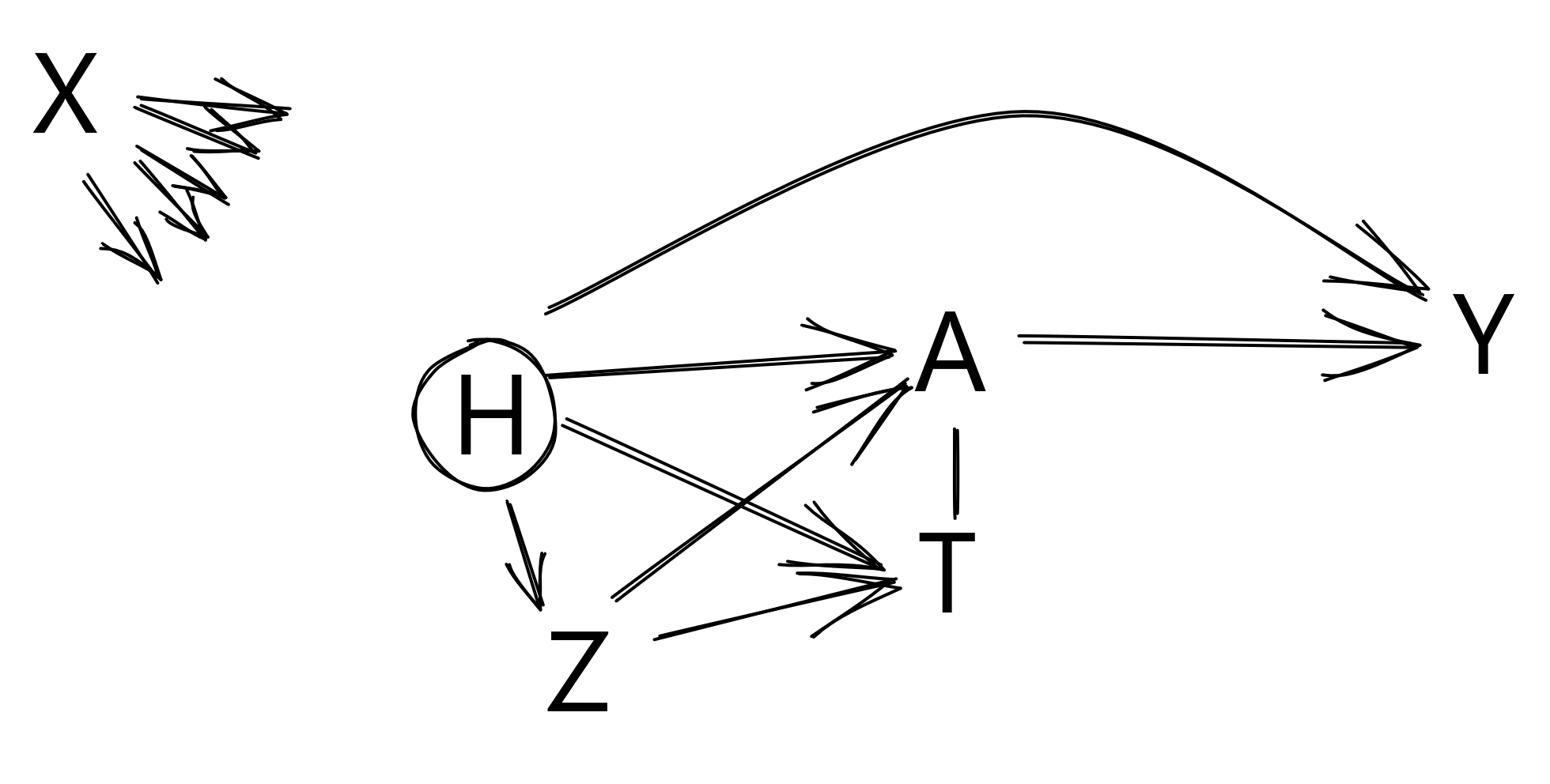


**Figure S1.** Causal diagram associated with the latent-variable model.

We used a latent-variable approach to evaluate admission versus discharge while adjusting for underlying latent patient health state and baseline characteristics. This approach has been detailed elsewhere (c.f., Cochran et al^2^); we will briefly describe it here. The approach involves building a model for the measured variables in the dataset and for an unmeasured or latent health state. The model consists of a probability distribution, up to unknown parameters, for the following random variables:

1. A vector *X* of baseline patient characteristics
2. A binary latent health state *H*
3. A vector *Z* of proxy variables that reflect initial noisy measurements of *H*
4. A binary admission decision *A*
5. A continuous treatment time *T* (i.e. time from treatment start to admission decision)
6. A binary outcome *Y*

The distribution for variables *(X, H, Z, A, T, Y)* is accompanied with the causal diagram depicted in Figure S1.

The causal diagram describes the following ED scenario. A patient with baseline characteristics *X* arrives to the ED in a certain unknown health state *H*. A health state of 1 signifies greater needs for clinical resources such as inpatient care. ED providers collect initial observations of the patient, including acuity and vitals (temperature, blood pressure, respiratory rate, and heart rate); these variables serve as proxies for the latent health state *H.* ED providers then begin to treat and monitor the patient, during which they accumulate additional evidence, beyond *Z*, of the latent health *H* through monitoring and testing until they have enough evidence for a patient with characteristics *X* to make their decision. The time of this decision is *T* and the decision itself is *A*. The patient is then either discharged or admitted according to *A*. Later we measure an outcome *Y* that reflects what decision *A* was made, the latent health state *H*, and possibly baseline characteristics *X*.

The probability distribution and the causal diagram constitute a causal model. That is, they induce a distribution on potential outcomes *Y(a)* that describe the outcome that would have been observed were the admission decision artificially set to be *a*. For the given causal diagram, potential outcomes can be interpreted as passing the quantity *a* forward instead of *A* along with passing the realized *H* and realized *X* in order to generate a random outcome. The latent-variable approach specifies a model for the distribution of Y as a function of *H, X*, and *A*, from which (by replacing *A* with *a*) the distribution of *Y(a)* can be recovered. In particular, the difference in the mean of *Y(1)* and *Y(0)* can be recovered; estimates of this risk difference, which adjusts for latent health state and baseline characteristics, is what is reported in the main text.

Note, the causal model embodies several standard assumptions for causal inference:

1. Well-defined potential outcomes *Y(a)*
2. Consistency: *Y(A) = Y*
3. Conditional exchangeability: *Y(a)* and *A* are independent conditional on *X* and *H*.

A final assumption, conditional positivity, is assumed, which says that the probability of being admitted or of being discharged, conditional on *X* and *H*, is strictly positive.

Learning the distribution for *Y(a)* rests on learning the distribution of *Y*. This is accomplished by specifying a distribution for all random variables, up to a set of unknown parameters. Health needs is linked to baseline characteristics by a logistic regression model. The specific proxy variable of acuity is linked to health state *H*, baseline characteristics *X*, and vitals by a logistic regression model. Other proxy variables *Z* are linked to health state *H* and baseline characteristics *X* by a linear regression model with normally distributed errors and unknown error variance. The admission process *(A,T)* is linked to health state *H*, baseline characteristics *X*, and initial observations *Z* by a survival analysis model known as threshold regression model.^3^ Specifically, the threshold regression model was a drift-diffusion process that hits one or two boundaries to reflect the decision-making scenario of the ED provider described above. For the threshold regression model, the boundary is linked to baseline characteristics *X* by a log-linear regression model; the starting point of the process, relative to the boundary, is linked to baseline characteristics *X* and proxy variables *Z* by a log-linear regression model; and the drift rate is linked to health state *H* by a linear regression model, but constrained in such a way that the drift rate is positive when *H=1* and negative when *H=0*. Last, the outcome *Y* is linked to patient characteristics *X* and latent health state *H* by a linear regression model; for simplicity, the regression terms for *X* are set to zero. In the end, the distribution for all random variables is defined up to a collection of unknown regression coefficients.

Unknown regression coefficients are estimated using maximum likelihood estimation via the expectation-maximization algorithm^4^ for each outcome. Confidence intervals (CIs) are estimated using a numerical approximation to Oakes Identity^5^ used in EM methods to estimate Fisher information matrix, which when inverted, yields an estimate of sampling variance for each parameter. These variances were then used to construct Wald 95% CIs assuming estimated parameters are normally distributed. CIs for functions of parameter estimates were estimated using the delta method. Significance was considered at a level of 0.05.

# **3-day and 9-day outcomes**

Secondary outcomes (3-day and 9-day outcomes) are summarized in Table S2. Estimates recovered from the latent variable approach are presented in Table S3. Unadjusted estimates, along with estimates for IPW and g-estimation, were previously presented for 3-day and 9-day outcomes in Table S1.

**Table S2.** Descriptive statistics of secondary outcomes by complaint.

|  | **All (N=3591)** | **Falls  (n=1581)** | **Weakness (n=564)** | **Syncope (n=468)** | **UTI (n=456)** | **Pneumonia (n=299)** | **Cellulitis (n=223)** |
| --- | --- | --- | --- | --- | --- | --- | --- |
|  | **Count (%)** | **Count (%)** | **Count (%)** | **Count (%)** | **Count (%)** | **Count (%)** | **Count (%)** |
| **3-day revisits** | 159 (4.4) | 72 (4.6) | 28 (5) | 11 (2.4) | 30 (6.6) | 9 (3) | 9 (4) |
| **9-day revisits** | 334 (9.3) | 145 (9.2) | 60 (10.6) | 24 (5.1) | 63 (13.8) | 20 (6.7) | 22 (9.9) |
| **3-day readmission** | 22 (0.6) | 3 (0.2) | 5 (0.9) | 3 (0.6) | 5 (1.1) | 5 (1.7) | 1 (0.4) |
| **9-day readmission** | 86 (2.4) | 16 (1) | 18 (3.2) | 6 (1.3) | 22 (4.8) | 16 (5.4) | 8 (3.6) |
| **3-day mortality** | 27 (0.8) | 6 (0.4) | 8 (1.4) | 0 (0) | 3 (0.7) | 9 (3) | 1 (0.4) |
| **9-day mortality** | 58 (1.6) | 18 (1.1) | 16 (2.8) | 2 (0.4) | 4 (0.9) | 17 (5.7) | 1 (0.4) |

**Table S3.** Adjusted estimates of risk difference (95% CI) between admission versus discharge for 3-day and 9-day outcomes adjusted for patient health state and measured patient variables.

| **Outcome** | **3-day revisits (%)** | **3-day readmission (%)** | **3-day mortality (%)** |
| --- | --- | --- | --- |
| **All** | -42.08 (-43.68,-40.48) | 0.61 (0.35,0.87) | -38.17 (-39.75,-36.60) |
| **Falls** | -25.17 (-27.30,-23.03) | 0.19 (-0.04,0.42) | 0.38 (0.04,0.71) |
| **Weakness** | 7.46 (-0.29,15.20) | 56.13 (52.18,60.08) | 56.66 (52.71,60.60) |
| **Syncope** | -27.98 (-57.56,1.61) | 10.88 (-18.74,40.51) | 0.00 (-0.14,0.14) |
| **UTI** | -4.39 (-6.71,-2.07) | 1.09 (0.14,2.05) | 0.66 (-0.09,1.41) |
| **Pneumonia** | 0.33 (-1.63,2.30) | 1.67 (0.21,3.12) | 2.34 (0.38,4.29) |
|  | **9-day revisits (%)** | **9-day readmission (%)** | **9-day mortality (%)** |
| **All** | -43.39 (-45.00,-41.78) | 2.39 (1.89,2.90) | 0.95 (0.53,1.36) |
| **Falls** | -28.14 (-30.35,-25.93) | 1.01 (0.48,1.54) | -20.80 (-22.79,-18.81) |
| **Weakness** | 6.49 (-1.07,14.04) | 58.43 (54.49,62.37) | 57.37 (53.43,61.32) |
| **Syncope** | -36.76 (-66.58,-6.94) | 4.27 (-30.23,38.77) | 0.43 (-0.17,1.02) |
| **UTI** | -56.74 (-61.25,-52.23) | 4.83 (2.85,6.80) | 0.88 (0.01,1.74) |
| **Pneumonia** | 4.01 (1.12,6.90) | 5.35 (2.80,7.90) | 19.06 (14.73,23.39) |

# **Sensitivity analyses**

Two additional analyses were performed with the intention of checking sensitivity of results to certain modeling decisions.

## ***Violations in conditional exchangeability***

First is to systematically violate the conditional exchangeability assumption embedded in the causal diagram for the latent variable approach. How these violations are conducted is detailed in Cochran et al.^2^ Results of this sensitivity analysis for 30-day outcomes across all diagnoses are presented in Table S4. The estimates for 30-day readmission and 30-day mortality are insensitive to violations of the conditional exchangeability assumption. The estimates for 30-day revisits are sensitivity to the specific violation, in which the probability of an admission for discharged patients is biased downwards by a factor of ψ_0_ = 0.95 for those who were to experience a 30-day revisit if discharged. The consequence of this violation, however, is such that the estimates presented in the main text are a more conservative estimate (i.e. closer to zero).

**Table S4.** Sensitivity analysis to violations in the identifiability assumption in the latent variable approach. This analysis includes all gray area diagnoses samples (N=3591).

| ψ_0_ | ψ _1_ | **30-Day Revisit, %** | **30-Day Readmission, %** | **30-Day Mortality, %** |
| --- | --- | --- | --- | --- |
| 1 | 1 | -6.43 (-7.80,-5.06) | 5.77 (5.00,6.53) | 1.03 (0.41,1.65) |
| 1 | 0.95 | -6.43 (-7.80,-5.06) | 5.77 (5.00,6.53) | 1.03 (0.42,1.64) |
| 1 | 0.975 | -6.43 (-7.80,-5.06) | 5.77 (5.00,6.53) | 1.03 (0.42,1.64) |
| 1 | 1.025 | -6.43 (-7.80,-5.06) | 5.77 (5.00,6.53) | 1.03 (0.41,1.65) |
| 1 | 1.05 | -6.43 (-7.80,-5.06) | 5.77 (5.00,6.53) | 1.03 (0.41,1.64) |
| 0.95 | 1 | -45.30 (-46.92,-43.67) | 5.77 (5.00,6.53) | 1.03 (0.42,1.64) |
| 0.95 | 0.95 | -45.30 (-46.92,-43.67) | 5.77 (5.00,6.53) | 1.03 (0.42,1.64) |
| 0.95 | 0.975 | -45.30 (-46.92,-43.67) | 5.77 (5.00,6.53) | 1.03 (0.42,1.64) |
| 0.95 | 1.025 | -45.30 (-46.92,-43.67) | 5.77 (5.00,6.53) | 1.03 (0.42,1.64) |
| 0.95 | 1.05 | -45.30 (-46.92,-43.67) | 5.77 (5.00,6.53) | 1.03 (0.42,1.65) |
| 0.975 | 1 | -6.42 (-7.79,-5.05) | 5.77 (5.00,6.53) | 1.03 (0.41,1.64) |
| 0.975 | 0.95 | -6.43 (-7.80,-5.06) | 5.77 (5.00,6.53) | 1.03 (0.42,1.64) |
| 0.975 | 0.975 | -6.43 (-7.80,-5.06) | 5.77 (5.00,6.53) | 1.03 (0.41,1.64) |
| 0.975 | 1.025 | -6.43 (-7.80,-5.06) | 5.77 (5.00,6.53) | 1.03 (0.42,1.64) |
| 0.975 | 1.05 | -6.42 (-7.79,-5.06) | 5.77 (5.00,6.53) | 1.03 (0.42,1.64) |
| 1.025 | 1 | -6.43 (-7.80,-5.06) | 5.77 (5.00,6.53) | 1.03 (0.42,1.64) |
| 1.025 | 0.95 | -6.43 (-7.80,-5.06) | 5.77 (5.00,6.53) | 1.03 (0.42,1.64) |
| 1.025 | 0.975 | -6.42 (-7.79,-5.05) | 5.77 (5.00,6.53) | 1.03 (0.42,1.65) |
| 1.025 | 1.025 | -6.42 (-7.79,-5.06) | 5.77 (5.00,6.53) | 1.03 (0.41,1.64) |
| 1.025 | 1.05 | -6.43 (-7.80,-5.06) | 5.77 (5.00,6.53) | 1.03 (0.42,1.64) |
| 1.05 | 1 | -6.43 (-7.80,-5.06) | 5.77 (5.00,6.53) | 1.03 (0.41,1.64) |
| 1.05 | 0.95 | -6.43 (-7.80,-5.06) | 5.77 (5.00,6.53) | 1.03 (0.41,1.64) |
| 1.05 | 0.975 | -6.42 (-7.79,-5.06) | 5.77 (5.00,6.53) | 1.03 (0.41,1.64) |
| 1.05 | 1.025 | -6.43 (-7.80,-5.06) | 5.77 (5.00,6.53) | 1.03 (0.42,1.64) |
| 1.05 | 1.05 | -6.42 (-7.79,-5.06) | 5.77 (5.00,6.53) | 1.03 (0.41,1.64) |

## ***Per-patient analysis***

Second is to check if a per-patient analysis, whereby each patient can only contribute once to the estimation, yields different estimates than the per-visit analysis presented in the main text. For a per-patient analysis, only the first ED visit is included in the sample; subsequent visits are excluded. The sample for per-patient analyses is summarized in Table S5. The latent variable approach was then applied to the new sample, resulting in the estimates reported in Table S6. The discrepancy in estimates between the two samples (i.e. per-patient sample and the per-visit sample) was not clinically meaningful: at most a 0.3% difference in the estimate. Therefore, the results from the primary analysis reported in the main text (i.e. latent variable approach) appear to be robust to correlation in data points due to repeated ED visits by the same person.

**Table S5.** Descriptive statistics of sample of older adult patients complaining of falls, weakness, syncope, UTI, pneumonia, and cellulitis and assigned an acuity level of 2 or 3 at a large ED in the Midwestern US (*per-patient analysis*).

|  | **All (N=3192)** | **Falls  (n=1330)** | **Weakness (n=512)** | **Syncope (n=445)** | **UTI (n=405)** | **Pneumonia (n=286)** | **Cellulitis (n=214)** |
| --- | --- | --- | --- | --- | --- | --- | --- |
|  | **Mean (SD)** | **Mean (SD)** | **Mean (SD)** | **Mean (SD)** | **Mean (SD)** | **Mean (SD)** | **Mean (SD)** |
| **Age, years** | 78.99 (8.93) | 80.22 (8.96) | 78.58 (8.49) | 76.78 (8.74) | 79.17 (8.89) | 78.94 (9.27) | 76.72 (8.5) |
| **Comorbidity, HCC** | 1.6 (1.41) | 1.446 (1.22) | 1.741 (1.432) | 1.266 (1.31) | 1.8 (1.58) | 2.081 (1.52) | 1.95 (1.73) |
| **Heart rate, BPM** | 78.38 (14.81) | 77.02 (13.69) | 78.38 (15.18) | 73.1 (13.68) | 80.61 (14.88) | 87.29 (15.96) | 81.66 (14.62) |
| **Temperature, °F** | 97.62 (1.45) | 97.45 (1.81) | 97.54 (0.86) | 97.35 (0.72) | 97.85 (1.19) | 98.59 (1.57) | 97.748 (0.81) |
| **Blood pressure, mmHg** | 74.11 (13.72) | 76.34 (13.98) | 74.34 (12.86) | 72.01 (12.18) | 73.44 (14.87) | 69.26 (13.48) | 71.879 (12.44) |
| **Respiration rate, BPM** | 18.1 (3.22) | 17.82 (2.949) | 17.89 (3.26) | 17.73 (3.58) | 18.25 (3.15) | 19.83 (3.76) | 18.43 (2.55) |
| **Treatment time, hours** | 0.18 (0.12) | 0.18 (0.1) | 0.2 (0.11) | 0.22 (0.2) | 0.16 (0.07) | 0.13 (0.06) | 0.15 (0.08) |
|  | **Count (%)** | **Count (%)** | **Count (%)** | **Count (%)** | **Count (%)** | **Count (%)** | **Count (%)** |
| **Female** | 1850 (58) | 818 (61.5) | 279 (54.5) | 241 (54.2) | 261 (64.4) | 141 (49.3) | 110 (51.4) |
| **Insurance** |  |  |  |  |  |  |  |
| Medicaid/Badger Care | 15 (0.5) | 3 (0.2) | 3 (0.6) | 5 (1.1) | 2 (0.5) | 1 (0.3) | 1 (0.5) |
| Medicare | 2905 (91.7) | 1226 (92.8) | 477 (93.5) | 394 (90.6) | 359 (89.1) | 261 (91.3) | 188 (87.9) |
| Commercial/Worker's Comp | 246 (7.8) | 92 (7) | 30 (5.9) | 34 (7.8) | 41 (10.2) | 24 (8.4) | 25 (11.7) |
| Self-Pay | 3 (0.1) | 0 (0) | 0 (0) | 2 (0.5) | 1 (0.2) | 0 (0) | 0 (0) |
| **Diabetes** | 651 (20.4) | 245 (18.4) | 120 (23.4) | 69 (15.5) | 90 (22.2) | 69 (24.1) | 58 (27.1) |
| **Congestive Heart failure** | 363 (11.4) | 143 (10.8) | 57 (11.1) | 30 (6.7) | 48 (11.9) | 50 (17.5) | 35 (16.4) |
| **Hypertension** | 1817 (56.9) | 756 (56.8) | 307 (60) | 230 (51.7) | 231 (57) | 162 (56.5) | 131 (61.2) |
| **Acuity = 2** | 902 (28.3) | 368 (27.7) | 127 (24.8) | 189 (42.5) | 94 (23.2) | 100 (35) | 24 (11.2) |
| **Admitted** | 1278 (40) | 287 (21.6) | 230 (44.9) | 168 (37.8) | 212 (52.3) | 243 (85) | 138 (64.5) |
| **3-day revisits** | 156 (4.9) | 69 (5.2) | 28 (5.5) | 11 (2.5) | 30 (7.4) | 9 (3.1) | 9 (4.2) |
| **9-day revisits** | 322 (10.1) | 138 (10.4) | 59 (11.5) | 24 (5.4) | 60 (14.8) | 19 (6.6) | 22 (10.3) |
| **30-day revisits** | 596 (18.7) | 264 (19.8) | 100 (19.5) | 51 (11.5) | 99 (24.4) | 38 (13.3) | 44 (20.6) |
| **3-day readmission** | 22 (0.7) | 3 (0.2) | 5 (1.0) | 3 (0.7) | 5 (1.2) | 5 (1.7) | 1 (0.5) |
| **9-day readmission** | 83 (2.6) | 16 (1.2) | 18 (3.5) | 6 (1.3) | 20 (4.9) | 15 (5.2) | 8 (3.7) |
| **30-day readmission** | 196 (6.1) | 47 (3.5) | 33 (6.4) | 23 (5.2) | 40 (9.9) | 31 (10.8) | 22 (10.3) |
| **3-day mortality** | 27 (0.8) | 6 (0.5) | 8 (1.6) | 0 (0) | 3 (0.7) | 9 (3.1) | 1 (0.5) |
| **9-day mortality** | 58 (1.8) | 18 (1.4) | 16 (3.1) | 2 (0.4) | 4 (1.0) | 17 (5.9) | 1 (0.5) |
| **30-day mortality** | 126 (3.9) | 49 (3.7) | 30 (5.9) | 6 (1.3) | 11 (2.7) | 27 (9.4) | 3 (1.4) |

**Table S6.** Risk difference (95% CI) of admission versus discharge on 9 outcomes adjusted for latent health state and measured patient variables (*per-patient analysis*).

| **Outcome** | **3-day revisits (%)** | **9-day revisits (%)** | **30-day revisits (%)** |
| --- | --- | --- | --- |
| **All** | -42.08 (-43.68,-40.48) | -43.39 (-45.01,-41.78) | -6.42 (-7.79,-5.06) |
| **Falls** | -25.11 (-27.25,-22.97) | -28.14 (-30.35,-25.93) | -33.15 (-35.47,-30.83) |
| **Weakness** | 7.46 (-0.29,15.20) | 6.49 (-1.07,14.04) | 4.38 (-2.84,11.60) |
| **Syncope** | -27.97 (-56.80,0.86) | -36.76 (-72.78,-0.74) | -38.65 (-43.24,-34.06) |
| **UTI** | -4.39 (-6.71,-2.07) | -56.74 (-61.25,-52.22) | -56.74 (-61.27,-52.21) |
| **Pneumonia** | 0.02 (-2.17,2.21) | 4.01 (1.12,6.90) | 23.41 (18.67,28.14) |
| **Cellulitis** | -66.50 (-72.50,-60.51) | -57.54 (-232.02,116.95) | 2.60 (-171.85,177.04) |
| **Outcome** | **3-day readmission (%)** | **9-day readmission (%)** | **30-day readmission (%)** |
| **All** | 0.61 (0.35,0.87) | 2.39 (1.89,2.89) | 5.77 (5.00,6.53) |
| **Falls** | 0.19 (-0.03,0.41) | 1.01 (0.51,1.51) | 3.16 (2.30,4.03) |
| **Weakness** | 56.13 (52.18,60.08) | 58.43 (54.49,62.37) | 61.62 (57.72,65.52) |
| **Syncope** | 10.90 (-18.81,40.61) | 4.27 (-33.86,42.41) | 4.92 (2.96,6.87) |
| **UTI** | 1.09 (0.06,2.13) | 4.83 (2.86,6.79) | 9.43 (6.75,12.11) |
| **Pneumonia** | 1.67 (0.20,3.14) | 5.35 (2.79,7.91) | 25.75 (20.92,30.58) |
| **Cellulitis** | 0.45 (-0.46,1.36) | 3.63 (1.15,6.11) | 28.00 (-130.75,186.75) |
| **Outcome** | **3-day mortality (%)** | **9-day mortality (%)** | **30-day mortality (%)** |
| **All** | -38.17 (-39.75,-36.60) | 0.95 (0.53,1.36) | 1.03 (0.41,1.64) |
| **Falls** | 0.38 (0.02,0.73) | -20.80 (-22.79,-18.81) | -0.89 (-1.76,-0.01) |
| **Weakness** | 56.66 (52.71,60.60) | 57.37 (53.42,61.32) | 58.08 (54.12,62.03) |
| **Syncope** | 0.00 (-0.07,0.07) | 0.43 (-0.17,1.02) | -21.95 (-53.39,9.49) |
| **UTI** | 0.66 (-0.10,1.41) | 0.88 (0.02,1.73) | 1.10 (-0.33,2.52) |
| **Pneumonia** | 2.34 (0.37,4.30) | 19.06 (14.75,23.36) | 7.69 (4.39,10.99) |
| **Cellulitis** | 0.45 (-0.48,1.38) | 0.45 (-0.45,1.35) | 1.36 (-0.37,3.09) |

# **Subgroup analyses**

The main text reported subgroup-specific estimates, recovered from the latent variable approach, for all diagnoses. **Table S7** presents these same subgroup-specific estimates but by individual diagnoses.

**Table S7.** Subgroup-specific estimates (95% CI) of risk difference between admission and discharge for 30-day outcomes, adjusted for latent health state and measured patient variables.

| **Diagnosis** | **Covariate** | **Group** | **30-day Revisit** | | **30-day Readmission** | | **30-day Mortality** | |
| --- | --- | --- | --- | --- | --- | --- | --- | --- |
|  |  |  | **Estimate (%)** | ***P*** | **Estimate (%)** | ***P*** | **Estimate (%)** | ***P*** |
| **All** | Age | 65-79.2 | -7.24 (-8.68,-5.79) | <.001 | 5.42 (4.68,6.17) | <.001 | 0.85 (0.24,1.45) | .01 |
|  |  | 79.2+ | -5.58 (-7.03,-4.13) |  | 6.12 (5.29,6.96) |  | 1.22 (0.58,1.86) |  |
|  | Sex | M | -6.45 (-7.96,-4.93) | <.001 | 5.76 (4.95,6.58) | <.001 | 1.03 (0.39,1.66) | <.001 |
|  |  | F | -6.42 (-7.86,-4.97) |  | 5.77 (4.98,6.55) |  | 1.03 (0.41,1.66) |  |
|  | Insurance | Medicare | -6.24 (-7.62,-4.87) | .001 | 5.84 (5.07,6.62) | .001 | 1.07 (0.45,1.69) | .001 |
|  |  | Other | -8.51 (-10.75,-6.26) |  | 4.88 (3.88,5.87) |  | 0.56 (-0.15,1.26) |  |
|  | Diabetes | No | -6.81 (-8.21,-5.41) | .96 | 5.60 (4.85,6.35) | .96 | 0.94 (0.33,1.55) | .96 |
|  |  | Yes | -4.98 (-6.72,-3.24) |  | 6.40 (5.44,7.36) |  | 1.36 (0.67,2.05) |  |
|  | Congenital Heart Failure | No | -7.03 (-8.42,-5.64) | .02 | 5.50 (4.77,6.24) | .02 | 0.89 (0.29,1.50) | .02 |
|  |  | Yes | -1.79 (-3.88,0.29) |  | 7.77 (6.57,8.98) |  | 2.09 (1.29,2.88) |  |
|  | Hypertension | No | -6.92 (-8.43,-5.40) | .01 | 5.56 (4.77,6.34) | .01 | 0.92 (0.30,1.54) | .01 |
|  |  | Yes | -6.08 (-7.52,-4.63) |  | 5.92 (5.11,6.72) |  | 1.11 (0.48,1.74) |  |
|  | HCC | ≤1.65 | -7.43 (-8.84,-6.02) | <.001 | 5.34 (4.61,6.06) | <.001 | 0.80 (0.20,1.40) | <.001 |
|  |  | >1.65 | -4.67 (-6.15,-3.18) |  | 6.52 (5.63,7.41) |  | 1.43 (0.77,2.09) |  |
| **Falls** | Age | 65-80.6 | -32.67 (-35.25,-30.09) | <.001 | 3.05 (2.18,3.93) | <.001 | -0.94 (-1.87,-0.02) | .03 |
|  |  | 80.6+ | -33.61 (-36.18,-31.03) |  | 3.27 (2.34,4.19) |  | -0.83 (-1.76,0.10) |  |
|  | Sex | M | -32.91 (-35.78,-30.05) | <.001 | 3.11 (2.17,4.04) | <.001 | -0.91 (-1.86,0.03) | .14 |
|  |  | F | -33.29 (-35.83,-30.76) |  | 3.20 (2.29,4.10) |  | -0.87 (-1.80,0.06) |  |
|  | Insurance | Medicare | -33.34 (-35.69,-30.99) | .41 | 3.21 (2.33,4.09) | .41 | -0.86 (-1.78,0.06) | .42 |
|  |  | Other | -30.82 (-35.78,-25.87) |  | 2.63 (1.38,3.87) |  | -1.17 (-2.25,-0.09) |  |
|  | Diabetes | No | -33.07 (-35.47,-30.67) | .78 | 3.14 (2.27,4.02) | .78 | -0.90 (-1.82,0.03) | .78 |
|  |  | Yes | -33.52 (-37.11,-29.92) |  | 3.25 (2.16,4.34) |  | -0.84 (-1.82,0.14) |  |
|  | Congenital Heart Failure | No | -32.33 (-34.69,-29.97) | .3 | 2.97 (2.15,3.80) | .3 | -0.99 (-1.90,-0.07) | .32 |
|  |  | Yes | -39.86 (-44.76,-34.97) |  | 4.71 (3.12,6.30) |  | -0.07 (-1.21,1.07) |  |
|  | Hypertension | No | -33.04 (-35.83,-30.25) | .8 | 3.14 (2.21,4.07) | .8 | -0.90 (-1.84,0.04) | .8 |
|  |  | Yes | -33.24 (-35.81,-30.67) |  | 3.18 (2.28,4.09) |  | -0.88 (-1.80,0.05) |  |
|  | HCC | ≤1.49 | -32.11 (-34.53,-29.69) | 0.002 | 2.92 (2.10,3.74) | .004 | -1.01 (-1.93,-0.10) | .01 |
|  |  | >1.49 | -34.94 (-37.66,-32.23) |  | 3.58 (2.56,4.59) |  | -0.67 (-1.62,0.29) |  |
| **Weakness** | Age | 65-78.7 | 10.99 (2.10,19.89) | .01 | 65.13 (60.45,69.80) | <.001 | 61.69 (56.90,66.49) | <.001 |
|  |  | 78.7+ | -2.82 (-12.18,6.53) |  | 57.81 (52.71,62.91) |  | 54.14 (48.98,59.30) |  |
|  | Sex | M | 1.03 (-9.01,11.07) | .35 | 59.89 (54.50,65.27) | <.001 | 56.25 (50.77,61.73) | <.001 |
|  |  | F | 7.35 (-2.08,16.77) |  | 63.16 (58.14,68.18) |  | 59.70 (54.57,64.83) |  |
|  | Insurance | Medicare | 3.31 (-4.11,10.73) | .02 | 61.08 (57.03,65.13) | **.02** | 57.63 (53.57,61.70) | .01 |
|  |  | Other | 20.59 (-8.48,49.67) |  | 69.83 (54.56,85.10) |  | 64.79 (50.88,78.70) |  |
|  | Diabetes | No | 8.38 (0.26,16.51) | .34 | 63.71 (59.40,68.02) | .35 | 60.27 (55.86,64.68) | .34 |
|  |  | Yes | -7.98 (-21.52,5.56) |  | 55.18 (47.90,62.45) |  | 51.32 (43.93,58.72) |  |
|  | Congenital Heart Failure | No | 7.67 (0.08,15.26) | .26 | 63.35 (59.30,67.40) | .28 | 59.91 (55.78,64.04) | .33 |
|  |  | Yes | -22.26 (-42.25,-2.28) |  | 47.62 (36.97,58.27) |  | 43.23 (32.46,54.00) |  |
|  | Hypertension | No | 3.03 (-7.94,14.00) | .04 | 60.91 (55.03,66.80) | .04 | 57.46 (51.46,63.46) | .03 |
|  |  | Yes | 5.20 (-3.66,14.07) |  | 62.06 (57.33,66.78) |  | 58.46 (53.64,63.27) |  |
|  | HCC | ≤1.82 | 12.82 (4.56,21.08) | .01 | 66.06 (61.74,70.39) | **.01** | 62.65 (58.19,67.11) | .003 |
|  |  | >1.82 | -10.07 (-20.09,-0.06) |  | 54.02 (48.52,59.53) |  | 50.26 (44.71,55.80) |  |
| **Syncope** | Age | 65-76.8 | -34.87 (-40.25,-29.49) | <.001 | 4.27 (2.49,6.04) | <.001 | -15.71 (-49.59,18.17) | .17 |
|  |  | 76.8+ | -43.30 (-48.86,-37.75) |  | 5.72 (3.39,8.06) |  | -29.66 (-59.28,-0.05) |  |
|  | Sex | M | -37.16 (-43.01,-31.32) | <.001 | 4.66 (2.70,6.63) | <.001 | -18.65 (-52.72,15.42) | .19 |
|  |  | F | -39.87 (-45.39,-34.34) |  | 5.13 (3.02,7.23) |  | -24.69 (-55.07,5.68) |  |
|  | Insurance | Medicare | -39.02 (-43.61,-34.42) | .01 | 4.98 (2.99,6.97) | .02 | -22.87 (-54.54,8.80) | .01 |
|  |  | Other | -35.14 (-48.70,-21.59) |  | 4.33 (1.62,7.05) |  | -13.43 (-52.60,25.73) |  |
|  | Diabetes | No | -37.46 (-42.32,-32.61) | .43 | 4.71 (2.81,6.61) | .44 | -20.07 (-52.45,12.32) | .3 |
|  |  | Yes | -44.25 (-52.88,-35.63) |  | 5.89 (3.23,8.54) |  | -30.90 (-61.55,-0.25) |  |
|  | Congenital Heart Failure | No | -37.12 (-41.78,-32.46) | .58 | 4.65 (2.79,6.52) | .59 | -19.64 (-52.02,12.74) | .46 |
|  |  | Yes | -58.61 (-71.67,-45.56) |  | 8.38 (4.51,12.26) |  | -52.26 (-81.84,-22.68) |  |
|  | Hypertension | No | -35.80 (-41.69,-29.91) | .14 | 4.43 (2.55,6.31) | .16 | -17.68 (-50.43,15.06) | .15 |
|  |  | Yes | -41.24 (-46.76,-35.71) |  | 5.36 (3.16,7.56) |  | -25.85 (-57.28,5.58) |  |
|  | HCC | ≤1.32 | -35.57 (-40.47,-30.68) | .001 | 4.39 (2.60,6.17) | .007 | -17.16 (-50.43,16.11) | .01 |
|  |  | >1.32 | -45.39 (-51.44,-39.34) |  | 6.09 (3.60,8.58) |  | -32.48 (-61.38,-3.58) |  |
| **UTI** | Age | 65-79.2 | -56.08 (-61.02,-51.14) | <.001 | 9.21 (6.51,11.91) | <.001 | 1.04 (-0.39,2.47) | .21 |
|  |  | 79.2+ | -57.51 (-62.56,-52.47) |  | 9.69 (6.84,12.54) |  | 1.16 (-0.30,2.63) |  |
|  | Sex | M | -57.58 (-63.09,-52.06) | <.001 | 9.71 (6.76,12.66) | <.001 | 1.17 (-0.31,2.65) | .1 |
|  |  | F | -56.26 (-61.16,-51.37) |  | 9.27 (6.56,11.98) |  | 1.05 (-0.38,2.49) |  |
|  | Insurance | Medicare | -56.89 (-61.48,-52.30) | .5 | 9.48 (6.78,12.19) | .5 | 1.11 (-0.33,2.55) | .51 |
|  |  | Other | -55.34 (-63.97,-46.70) |  | 8.96 (5.41,12.51) |  | 0.97 (-0.57,2.52) |  |
|  | Diabetes | No | -55.71 (-60.44,-50.97) | .61 | 9.09 (6.46,11.71) | .61 | 1.01 (-0.41,2.42) | .61 |
|  |  | Yes | -60.47 (-66.79,-54.15) |  | 10.69 (7.33,14.05) |  | 1.43 (-0.14,2.99) |  |
|  | Congenital Heart Failure | No | -55.87 (-60.51,-51.22) | .71 | 9.14 (6.52,11.76) | .71 | 1.02 (-0.40,2.44) | .71 |
|  |  | Yes | -62.98 (-70.59,-55.36) |  | 11.53 (7.70,15.36) |  | 1.65 (-0.03,3.32) |  |
|  | Hypertension | No | -54.65 (-59.99,-49.31) | .11 | 8.73 (6.07,11.40) | .11 | 0.91 (-0.50,2.33) | .15 |
|  |  | Yes | -58.38 (-63.36,-53.41) |  | 9.99 (7.07,12.90) |  | 1.24 (-0.24,2.72) |  |
|  | HCC | ≤1.88 | -54.74 (-59.56,-49.93) | .05 | 8.76 (6.20,11.32) | .06 | 0.92 (-0.48,2.32) | .09 |
|  |  | >1.88 | -60.43 (-65.59,-55.27) |  | 10.67 (7.55,13.80) |  | 1.42 (-0.11,2.95) |  |
| **Pneumonia** | Age | 65-78.9 | 27.18 (21.48,32.87) | <.001 | 30.36 (24.33,36.39) | <.001 | 6.93 (3.65,10.21) | <.001 |
|  |  | 78.9+ | 19.46 (14.56,24.35) |  | 20.91 (15.65,26.17) |  | 8.49 (5.07,11.91) |  |
|  | Sex | M | 24.32 (18.80,29.84) | <.001 | 26.87 (20.91,32.82) | <.001 | 7.50 (4.19,10.82) | <.001 |
|  |  | F | 22.49 (17.14,27.84) |  | 24.62 (18.83,30.42) |  | 7.87 (4.52,11.23) |  |
|  | Insurance | Medicare | 22.02 (17.31,26.74) | .002 | 24.06 (19.13,28.98) | .002 | 7.97 (4.64,11.30) | .01 |
|  |  | Other | 38.55 (25.11,51.99) |  | 44.28 (28.63,59.92) |  | 4.64 (0.54,8.74) |  |
|  | Diabetes | No | 23.91 (18.90,28.91) | .51 | 26.36 (21.11,31.60) | .51 | 7.59 (4.29,10.88) | .51 |
|  |  | Yes | 21.83 (15.44,28.22) |  | 23.82 (16.59,31.05) |  | 8.01 (4.56,11.45) |  |
|  | Congenital Heart Failure | No | 24.51 (19.53,29.48) | .01 | 27.09 (21.92,32.26) | .01 | 7.47 (4.19,10.75) | .03 |
|  |  | Yes | 18.31 (12.07,24.54) |  | 19.51 (12.40,26.61) |  | 8.72 (5.17,12.26) |  |
|  | Hypertension | No | 25.62 (19.73,31.52) | .5 | 28.46 (22.06,34.86) | .5 | 7.24 (3.92,10.56) | .5 |
|  |  | Yes | 21.73 (16.61,26.85) |  | 23.69 (18.17,29.21) |  | 8.03 (4.67,11.39) |  |
|  | HCC | ≤2.11 | 23.96 (18.89,29.02) | .04 | 26.42 (21.09,31.75) | .04 | 7.58 (4.28,10.88) | .06 |
|  |  | >2.11 | 22.56 (17.20,27.93) |  | 24.72 (18.90,30.53) |  | 7.86 (4.51,11.21) |  |

*Note. P*-values are reported for a Wald hypothesis test, checking for significant moderation of each patient characteristic.

# **Cellulitis**

Older adult patients with cellulitis had the smallest sample and yielded imprecise estimates. Thus, we reported results for cellulitis here. For these patients, admission carried a numerically higher risk than a discharge for 30-day revisits, readmission, and mortality, but these estimates were not significant (Table S8). Estimated risks for 3- and 9-day revisits were not consistent with 30-day results, with admission significantly lowering 3-day revisit risks by an estimated adjusted average of 66.51% (95% CI: [60.52, 72.5]). Admission also carried a lower 9-day revisit risk, but the estimate was not significant. Admission increased risks of 3- and 9-day readmission, but only the 9-day readmission risk was significant (3.63% [1.15, 6.11]). Admission was also estimated to increase the risks of for 3-day and 9-day mortality, but these estimates were not significant.

Sub-group specific estimates are provided in Table S9. In part because of the imprecise estimates for the cellulitis group, none of the patient characteristics were significant moderators of the adjusted risk difference. Unadjusted estimates and estimates recovered from IPW and g-estimation are presented in Table S10. The direction of these estimates agreed with the those of other individual diagnoses: negative for revisits and positive for readmissions and mortality.

**Table S8.** Estimated risk difference (95% CI) between admission versus discharge for cellulitis diagnosis adjusted for latent health state and measured patient variables.

|  | **3-day (%)** | **9-day (%)** | **30-day (%)** |
| --- | --- | --- | --- |
| **Revisits** | -66.51 (-72.50,-60.52) | -57.60 (-224.56, 109.37) | 2.60 (-163.64, 168.84) |
| **Readmission** | 0.45 (-0.46, 1.36) | 3.63 (1.15, 6.11) | 28.02 (-110.20, 166.23) |
| **Mortality** | 0.45 (-0.46, 1.36) | 0.45 (-0.51, 1.41) | 1.36 (-0.36, 3.08) |

**Table S9.** Among patients with a cellulitis diagnosis, subgroup-specific estimates of risk difference (95% CI) between admission and discharge for 30-day outcomes, adjusted for latent health state and measured patient variables.

| **Diagnosis** | **Covariate** | **Group** | **30-day Revisit** | | **30-day Readmission** | | **30-day Mortality** | |
| --- | --- | --- | --- | --- | --- | --- | --- | --- |
|  |  |  | **Estimate (%)** | **p-val** | **Estimate (%)** | **p-val** | **Estimate (%)** | **p-val** |
| **Cellulitis** | Age | 65-76.8 | 3.31 (-165.44,172.05) | .96 | 28.86 (-118.55,176.26) | .72 | 1.31 (-0.36,2.97) | .13 |
|  |  | 76.8+ | 1.73 (-162.16,165.62) |  | 26.99 (-99.95,153.93) |  | 1.42 (-0.38,3.22) |  |
|  | Sex | M | 2.63 (-163.50,168.76) | .99 | 28.06 (-110.22,166.34) | .67 | 1.35 (-0.37,3.07) | .12 |
|  |  | F | 2.56 (-163.80,168.93) |  | 27.98 (-110.21,166.17) |  | 1.36 (-0.37,3.09) |  |
|  | Insurance | Medicare | 1.90 (-162.11,165.91) | .89 | 27.20 (-101.65,156.04) | .86 | 1.41 (-0.37,3.18) | .41 |
|  |  | Other | 7.67 (-188.50,203.84) |  | 33.99 (-172.41,240.39) |  | 1.02 (-0.35,2.38) |  |
|  | Diabetes | No | 3.51 (-166.17,173.19) | .97 | 29.09 (-120.94,179.13) | .97 | 1.29 (-0.35,2.94) | .94 |
|  |  | Yes | 0.17 (-161.13,161.48) |  | 25.16 (-81.74,132.07) |  | 1.53 (-0.41,3.46) |  |
|  | Congenital Heart Failure | No | 3.86 (-167.37,175.10) | .89 | 29.51 (-125.12,184.15) | .86 | 1.27 (-0.35,2.88) | .24 |
|  |  | Yes | -3.99 (-171.70,163.73) |  | 20.27 (-32.94,73.47) |  | 1.82 (-0.47,4.11) |  |
|  | Hypertension | No | 3.29 (-165.45,172.04) | .88 | 28.85 (-118.57,176.26) | .86 | 1.31 (-0.36,2.98) | .26 |
|  |  | Yes | 2.18 (-162.81,167.17) |  | 27.52 (-105.17,160.20) |  | 1.39 (-0.37,3.15) |  |
|  | HCC | ≤1.97 | 4.41 (-169.39,178.20) | .88 | 30.15 (-131.55,191.85) | .86 | 1.23 (-0.34,2.80) | .15 |
|  |  | >1.97 | -0.34 (-161.50,160.83) |  | 24.56 (-75.58,124.70) |  | 1.56 (-0.41,3.54) |  |

**Table S10.** Comparison of unadjusted estimates of risk differences, IPW estimates, and g-estimation estimates (95% CI) for cellulitis diagnosis.

| **Diagnosis** | **Outcome** | **Unadjusted** | **IPW** | **G-estimation** |
| --- | --- | --- | --- | --- |
| **Cellulitis** | **3-day Revisit** | -9.2 (-11.5, -6.9) | -8.5 (-11.4, -5.7) | -10.0 (-10.2, -3.5) |
|  | **9-day Revisit** | -11.7 (-15.1, -8.2) | -17.0 (-25.7, -8.2) | -15.1 (-15.4, -6.4) |
|  | **30-day Revisit** | -12.9 (-17.6, -8.2) | -15.1 (-27.6, -2.5) | -16.0 (-16.4, -4.4) |
|  | **3-day Readmission** | 0.7 (-0.1, 1.5) | 0.6 (0.4, 0.8) | 0.6 (-0.0, 2.7) |
|  | **9-day Readmission** | 5.6 (3.5, 7.8) | 4.7 (3.8, 5.6) | 3.9 (3.7, 8.8) |
|  | **30-day Readmission** | 15.5 (12.0, 19.0) | 17.3 (11.7, 23.0) | 14.3 (14.0, 21.9) |
|  | **3-day Mortality** | 0.7 (-0.1, 1.5) | 0.5 (0.4, 0.7) | 0.4 (-0.0, 2.7) |
|  | **9-day Mortality** | 0.7 (-0.1, 1.5) | 0.5 (0.4, 0.7) | 0.4 (-0.0, 2.7) |
|  | **30-day Mortality** | 2.1 (0.8, 3.5) | 1.9 (1.3, 2.5) | 2.0 (-0.0, 6.0) |

# **Syndromic and no clear standard practice diagnoses**

A final analysis is performed where we group diagnoses based on whether they are syndromic (falls and weakness) or whether there is no clear standard of practice (syncope, UTI, and pneumonia). Results from latent variable approach for these two groups are presented in **Table S11**.

**Table S11.** Adjusted estimates (95% CI) of risk difference between admission versus discharge for 9 outcomes adjusted for latent health state and measured patient variables. Patients are group based on whether their diagnosis was syndromic diagnoses or had no clear standard practice. “Syndromic diagnoses” include falls and weakness population (n=2145). “No clear standard practice diagnoses” include syncope, UTI, pneumonia, and cellulitis (n=1223).

| **Outcome** | **Syndromic diagnoses** | **No clear standard practice diagnoses** |
| --- | --- | --- |
| **3-day revisits (%)** | 41.59 (37.88,45.30) | -1.98 (-3.11,-0.84) |
| **9-day revisits (%)** | -33.56 (-35.54,-31.57) | -56.46 (-59.23,-53.70) |
| **30-day revisits (%)** | -37.85 (-39.90,-35.81) | -54.92 (-57.71,-52.14) |
| **3-day readmission (%)** | 0.37 (0.11,0.63) | 1.06 (0.48,1.64) |
| **9-day readmission (%)** | 1.58 (1.06,2.11) | 3.60 (2.55,4.64) |
| **30-day readmission (%)** | 4.01 (3.18,4.84) | 8.10 (6.57,9.63) |
| **3-day mortality (%)** | 0.65 (0.30,1.00) | -54.07 (-56.84,-51.30) |
| **9-day mortality (%)** | -26.55 (-28.41,-24.69) | -53.34 (-56.11,-50.56) |
| **30-day mortality (%)** | 0.09 (-0.72,0.91) | 2.61 (1.56,3.67) |

# **References**

1. Wooldridge JM. *Econometric Analysis of Cross Section and Panel Data*. MIT press; 2010.

2. Cochran A, Rathouz PJ, Kocher KE, Zayas-Caban G. A latent variable approach to potential outcomes for emergency department admission decisions. *Statistics in Medicine*. Published online accepted 2019. doi:10.1002/sim.8210

3. Lee MLT, Whitmore GA. Threshold Regression for Survival Analysis: Modeling Event Times by a Stochastic Process Reaching a Boundary. *Statistical Science*. 2006;21(4):501-513.

4. Dempster AP, Laird NM, Rubin DB. Maximum likelihood from incomplete data via the EM algorithm. *Journal of the Royal Statistical Society: Series B (Methodological)*. 1977;39(1):1-22.

5. Oakes D. Direct calculation of the information matrix via the EM. *Journal of the Royal Statistical Society: Series B (Statistical Methodology)*. 1999;61(2):479-482.
